# Need for Cognitive Closure Modulates How Perceptual Decisions Are Affected by Task Difficulty and Outcome Relevance

**DOI:** 10.1371/journal.pone.0146002

**Published:** 2015-12-30

**Authors:** Vanda Viola, Annalisa Tosoni, Ambra Brizi, Ilaria Salvato, Arie W. Kruglanski, Gaspare Galati, Lucia Mannetti

**Affiliations:** 1 Department of Psychology, University of Rome ''La Sapienza'', Rome, Italy; 2 Laboratory of Neuropsychology, Foundation Santa Lucia, Rome, Italy; 3 Department of Neuroscience, Imaging and Clinical Science, University G. D’Annunzio and Institute for Advanced Biomedical Technology, Foundation G. D’Annunzio, Chieti, Italy; 4 Department of Developmental and Social Psychology, University of Rome ''La Sapienza'', Rome, Italy; 5 Department of Psychology, University of Maryland, College Park, Maryland, United States of America; ghent university, BELGIUM

## Abstract

The aim of this study was to assess the extent to which Need for Cognitive Closure (NCC), an individual-level epistemic motivation, can explain inter-individual variability in the cognitive effort invested on a perceptual decision making task (the random motion task). High levels of NCC are manifested in a preference for clarity, order and structure and a desire for firm and stable knowledge. The study evaluated how NCC moderates the impact of two variables known to increase the amount of cognitive effort invested on a task, namely task ambiguity (i.e., the difficulty of the perceptual discrimination) and outcome relevance (i.e., the monetary gain associated with a correct discrimination). Based on previous work and current design, we assumed that reaction times (RTs) on our motion discrimination task represent a valid index of effort investment. Task ambiguity was associated with increased cognitive effort in participants with low or medium NCC but, interestingly, it did not affect the RTs of participants with high NCC. A different pattern of association was observed for outcome relevance; high outcome relevance increased cognitive effort in participants with moderate or high NCC, but did not affect the performance of low NCC participants. In summary, the performance of individuals with low NCC was affected by task difficulty but not by outcome relevance, whereas individuals with high NCC were influenced by outcome relevance but not by task difficulty; only participants with medium NCC were affected by both task difficulty and outcome relevance. These results suggest that perceptual decision making is influenced by the interaction between context and NCC.

## Introduction

In everyday life people are continuously asked to choose between investing cognitive effort in demanding tasks or saving resources by adopting less effortful cognitive strategies. In some instances we make deliberate, explicit choices between two active tasks (e.g. studying for an exam vs. watching a movie) but much more frequently choices about the level of cognitive effort invested in a single task are made unreflectively or even unconsciously. These choices may have implications for both the decision maker (e.g. future career) and the safety of his or her community (e.g. air traffic control, power plant operation). In social cognition “thinking” has been rightly conceived as “work” and people’s reluctance to invest cognitive effort has led to the use of terms such as “cognitive miser” and “motivated tactician” [1]. Recently, individuals’ willingness to invest cognitive effort has been studied using adaptations of behavioral economic paradigms in which preferences are inferred from choice behavior rather than from self-reports [2]. In these research tasks participants are asked to choose between a low-effort task associated with a small monetary reward and a high-effort task associated with a larger reward; the results show that there is considerable inter-individual variability in the perceived cost of cognitive effort [2]. This recent literature has also highlighted the importance of taking into account individual differences in willingness to invest cognitive effort, as they may have important implications for the prediction and explanation of everyday behavior and the design and implementation of strategies to stimulate greater cognitive effort in educational and employment contexts.

In this study we examined the association between Need for Cognitive Closure (NCC), an individual-level epistemic motivation that is manifested as a preference for clarity, order and structure and a desire for firm and stable knowledge [3], and the cognitive effort invested in a perceptual decision task. More precisely, we assessed whether NCC moderates the impact of two factors traditionally thought to increase cognitive effort: task difficulty and outcome relevance. In the following paragraphs we provide a brief review of research on these variables and on NCC.

### Task Difficulty, Outcome Relevance and Cognitive Effort

According to the Motivational Intensity Theory [4], [5], the intensity of cognitive effort invested in a task depends on the perceived difficulty of the task and the reward associated with successful performance (i.e. outcome relevance). The Motivational Intensity Theory follows the difficulty law of motivation [6], [7], in assuming that cognitive effort normally increases as a function of task difficulty, but stops increasing when the task is perceived as too difficult to complete successfully (i.e. excessive task difficulty triggers disengagement). Outcome relevance, the second most important determinant of cognitive effort according to Motivational Intensity Theory, is defined as the importance of successful performance to the individual performer; the more important success on a task is to a given individual, the more effort he or she is justified in expending on it. In summary, the central prediction of the Motivational Intensity Theory is that task difficulty and outcome relevance influence cognitive effort. Several studies have identified psychological variables which influence both perceived task difficulty and outcome relevance. In particular, perceived task difficulty has been linked to task demand (e.g., [8]) and perceived fatigue (e.g., [9]), [10] whereas outcome relevance has been shown to be influenced by the individual’s current need state (e.g., [11]), performance-contingent reward (e.g., [12]),[13] and performance-contingent avoidance of noxious stimulation [14].

### 1.2. Need for Cognitive Closure and its Impact on Cognitive Processes

Need for closure has been defined as individuals’ ‘desire for a firm answer to a question, any firm answer as compared to confusion and/or ambiguity’ ([5], p. 15) and is assumed to vary along a continuum ranging from strong need for closure to strong need to avoid closure. A strong need for closure is experienced as an urgent desire for permanent closure; individuals with a strong need for closure tend to “seize” on information which allows them to make a judgment on a given topic and then “freeze” on that judgment, becoming relatively impermeable or close-minded to further relevant information [3]. Such individuals also make strong commitments and are relatively unshakeable in their views. In contrast individuals with a strong need to avoid closure are wary of commitments, feel more comfortable keeping their options open and eschew binding or definite opinions. Importantly, an individual’s position on the need for closure continuum is determined by how they perceive the relative benefits and costs of gaining or not gaining closure, and this is determined by internal factors (dispositional NCC) and by contextual features such as time pressure, boredom, noise and fatigue (situation-induced NCC, for a review see [15]).

It is worth pointing out that NCC is not, as Cacioppo and Petty [16] proposed, simply the opposite of need for cognition. Need for cognition is defined as the amount of effortful cognitive activity that an individual seeks and enjoys [16]. Individuals with high need for cognition process information in a more elaborate and effortful manner [17]. As Webster and Kruglanski [18] made clear, need for cognition influences the quantity of cognitive activity one engages in, thus determining the effort an individual will invest in a task, whereas need for closure relates to the motivation underlying cognitive effort. This means that need for cognition, and consequently cognitive effort are reduced by NCC.

As a consequence, the relation between the need for cognition and the need for cognitive closure is not straightforward (see [18]). Indeed, while people reporting high need for cognition process information in more effortful and elaborative way [17], the need for cognitive closure is defined by the will to reach a definite answers as soon as possible.

Kruglanski and colleagues [19] recently proposed a Cognitive Energetic Theory (CET) which uses field-force analysis to provide insight into the mechanisms underlying selection of cognitive effort level for a task. CET, which builds on previous theories such Motivation Intensity Theory [4], [5] and the Lay Epistemic Theory [20], posits that at the moment of choice there are forces driving cognitive effort (e.g. goal importance and resource availability), and forces restraining cognitive effort (e.g. task demand and personal tendency to conserve cognitive resources, i.e. NCC). Unlike Motivation Intensity Theory [4], [5], which focuses on objective modulators of effort, the Lay Epistemic Theory [20] focuses on individual factors and CET integrates these two theories by considering both factors together.

Overall both the NCC literature and the new CET predict that high NCC individuals will be less motivated to invest cognitive effort in a task than low NCC individuals. This prediction has been confirmed by recent research on the neural mechanisms underlying NCC [21], [22]. In particular, Viola et al. [21] recently showed that relatively high NCC is associated with reduced online adjustment in cognitive control, as indexed by adaptation to behavioral conflict. This behavioral effect appeared to be mediated by dynamic changes in cortico-cortical functional connectivity between prefrontal regions involved in conflict monitoring and implementation of cognitive control. Specifically, after exposure to conflict functional connectivity appeared to be increased in low NCC but not high NCC individuals [21]. Moreover, in a recent study using event-related potentials Kossowska and colleagues [22] showed that NCC reflected basic differences in conflict monitoring, demonstrating that greater NCC predicted lower levels of conflict-related activity. Both these studies support the hypothesis that there is a negative relationship between NCC and cognitive effort.

Drawing on this recent literature, the objectives of this study were to confirm the negative correlation between NCC and cognitive effort and to assess whether NCC moderates the influence of task difficulty and outcome relevance on cognitive effort [4],[5].

To our knowledge only one previous study has investigated the interaction between NCC, task difficulty and cognitive effort [23] and the results showed that task difficulty only increased cognitive effort in conditions of situationally-induced low NCC (but not in conditions of situationally-induced high NCC).

This study was intended to extend the results of the earlier research in two ways. Firstly, we intended to assess whether the effects obtained by Roets and colleagues [23] using a situational manipulation of NCC could be replicated with respect to dispositional NCC.

More in particular, we tested for possible interactions between dispositional and situational factors, where dispositional factors are defined as personal and individual tendencies that applies generally across different situations in contrast to manipulations of external conditions that affect the individual behavior (situational factors)

Here we address this question by having subjects with different dispositional NCC levels (as indicated by scores on the Need for Cognitive Closure Scale [24]) perform a motion discrimination task in which situational factors were manipulated through a modulation of task difficulty obtained by varying the ambiguity level of the motion stimuli.

Secondly, we wanted to assess whether dispositional NCC moderated the influence of outcome relevance—operationalized as the monetary gain associated with correct performance—as well as moderating the impact of task difficulty. We followed an earlier study [23] in indexing cognitive effort using reaction time (RT) measures, based on the assumption that higher RTs during our motion discrimination task represent a valid index of effort investment intended as a willingness to spend time viewing the motion display.

Following theories, which posit that low NCC individuals have intrinsic motivations to invest cognitive effort [25], [26], we predicted that changes in task difficulty (operationalized as stimulus ambiguity) would bring low NCC participants to spend more time before making a decision. Conversely, we hypothesized that increasing outcome relevance (operationalized as the monetary reward for a correct discrimination) would increase RTs in high but not low NCC participants. This hypothesis was based on the assumptions that high NCC individuals perceive cognitive effort as less useful than low NCC individuals and are as sensitive to monetary rewards as individuals whose mental resources are exhausted [27], [28]. The cognitive effort can be defined as the mental effort spent by an individual on a process [29] and on this basis there are many ways in which cognitive effort can be operazionalized in addition or in alternative to RTs on a task. For example, larger gaze time have been taken to be a direct reflection of cognitive effort [30], [31] but if it is requested a response selection between two competitive options also RTs are expected to be longer in condition of high cognitive effort demand (e.g. [29]). In another series of studies by Roets et al. [23] in which participants with low and high levels of NFC (dispositional or situational/manipulated) performed a cognitive task with different levels of difficulty. In these studies the cognitive effort was measured as participants’ information-gathering behavior and by self-report measures of the perceived effort investment. The results showed a positive correlation between invested effort and cognitive closure only for individuals with low NFC scores. These results foster the expectations that the NFC influences the effort that people are prepared to invest.

## Method

### 2.1. Subjects

Three hundred and seventy-three volunteers (18-36 years old) completed a questionnaire about decisional styles and the Need for Cognitive Closure Scale (NCCS; [24]). The sample was divided into three sub-groups based on NCC score (high NCC: 4th quartile, medium NCC: 2nd and 3rd quartile, low NCC: 1st quartile) and we recruited participants with scores as near as possible to the median of each sub-group. Twenty-one subjects with low NCC (mean NCC = 34.71, *SD* = 3.50), 20 subjects with medium NCC (mean NCC = 42.27 *SD* = 2.78), and 19 subjects with high NCC (mean NCC = 53.94, *SD* = 5.49) agreed to participate in the behavioral experiment. All participants had normal or corrected-to-normal vision and gave written informed consent to participation. The study was approved by the Ethics Committee of the Santa Lucia Foundation (Scientific Institute for Research Hospitalization and Health Care). The participants were fully debriefed about the aims of the study after the experiments.

### 2.2. Random Dot Motion (RDM) Stimuli and Task

The random dot motion (RDM) task [32–45] is a classical perceptual decision making task. The stimuli are clouds of dots presented on a computer screen; a certain percentage of dots in a cloud move in a specific direction (the target direction) whilst the remainder move randomly. Participants are required to indicate the apparent direction of the dot cloud; the percentage of dots moving in the target direction is typically used as a measure of task difficulty (see e.g. [34]). In order to respond correctly, an observer should count the number of dots moving in each direction; however the motion-sensitive neurons in the brain respond to their preferred motion direction and to similar directions, so the perceptual system introduces variance to the perceived motion and decisions are typically made on the basis of overall perceptual impression.

Stimuli were generated using in-house software, implemented in MATLAB (The MathWorks Inc., Natick, MA; USA) [46] using Cogent 2000 [47] (developed at FIL and ICN, UCL, London, UK) and Cogent Graphics (developed by John Romaya at LON, Wellcome Department of Imaging Neuroscience, UCL, London, UK). The motion stimulus was a random-dot kinetogram [34] contained within a circular aperture with 0.75° diameter centered on a fixation point. The dots were white squares subtending 0.15° and were displayed against a black background. The dots were plotted in three interleaved sets of equal size. Each set was plotted in one of three successive video frames (frame rate = 75Hz) and shown for one frame. Three frames (40ms) later a proportion of the dots from the set was plotted at a displacement of 0.2° to give the impression of coherent motion and the remaining dots were re-plotted at random locations. Together, the three sets produced a dot density of 50° per second. A fixation cross appeared at the centre of the display throughout each trial.

### 2.3. Psychophysical Calibration Session

**Fig 1:**
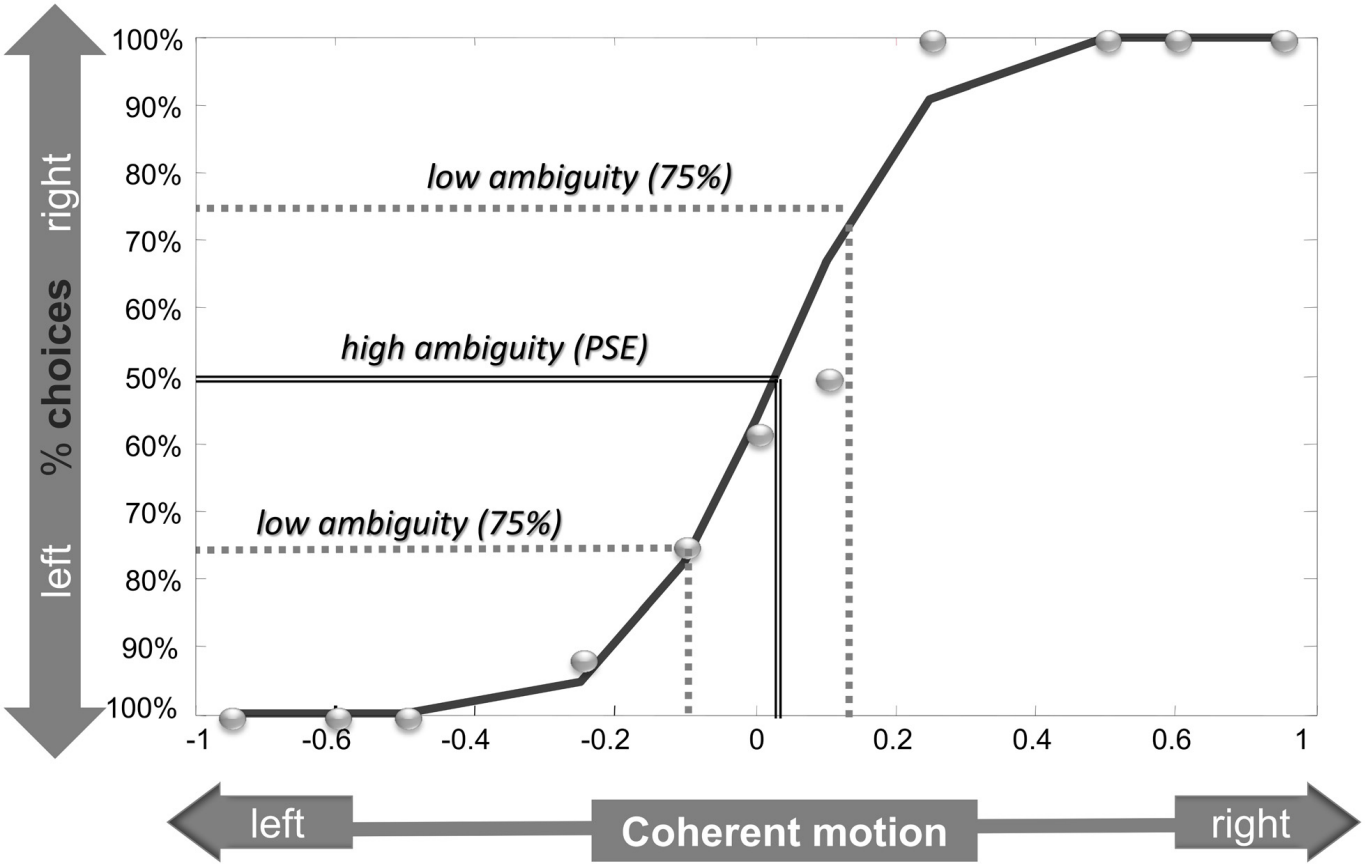
Psychophysical calibration session. The graphs illustrate the selection of motion coherence levels yielding a rightwards response in 25% (low ambiguity), 50% (high ambiguity) and 75% (low ambiguity) of cases. The solid curve represents the best-fitting psychometric function for a representative subject, and describes the probability of a rightwards response as a function of the motion coherence of the RDM stimulus. The scatter plot shows the raw data from which the estimate was computed.

Before the main experiment we used the RDM direction discrimination task to determine two motion coherence levels for each subject, one associated with chance discrimination levels (high ambiguity; difficult decision) and one associated with a 75% probability of selecting a given (left or right) direction of motion (low ambiguity; easy decision) (Fig 1). Eleven motion coherence levels (percentage of stimuli moving consistently in a given direction: 0% and 10%, 25%, 50%, 60%, 75% for both leftwards and rightward motion) were presented in this calibration session; all the levels were presented 12 times and levels were randomly interleaved from trial to trial. The RDM stimuli were 132 dot clouds depicted in green and were presented for a random interval (range: 100-1500ms). Each trial started with a 1000ms presentation of a static cloud; this was followed by a 5000ms presentation of a moving cloud.

Calibration data were subjected to a probit analysis of binomial responses based on maximum likelihood estimation [48], [49]; this allowed us to determine the threshold and slope of a psychometric function describing the probability of choosing a given motion direction as a function of motion coherence for individual subjects. Interpolation of this psychometric function was used to select the motion coherence level which would yield a given directional response 50% or 75% of the time on average (Fig 1). The 50% level (point of subjective equality; PSE) was used as the high ambiguity condition and the 25% and 75% levels were used as low ambiguity condition for the two directions. Five subjects were excluded after the calibration phase because their psychophysical curves were a bad fit to the psychometric function (*r* ^2^ < .50).

It is important to note that using individually calibrated motion coherence levels provided a degree of control for inter-subject variability in motion perception and motion classification without affecting potential RT variability in response to task manipulations.

### 2.4. Main Experiment

The main task consisted of 72 experimental trials: 48 low ambiguity trials (24 for each motion direction) and 24 high ambiguity trials. The coherence levels for the low and high ambiguity trials were individually selected during the calibration phase as described above.

**Fig 2:**
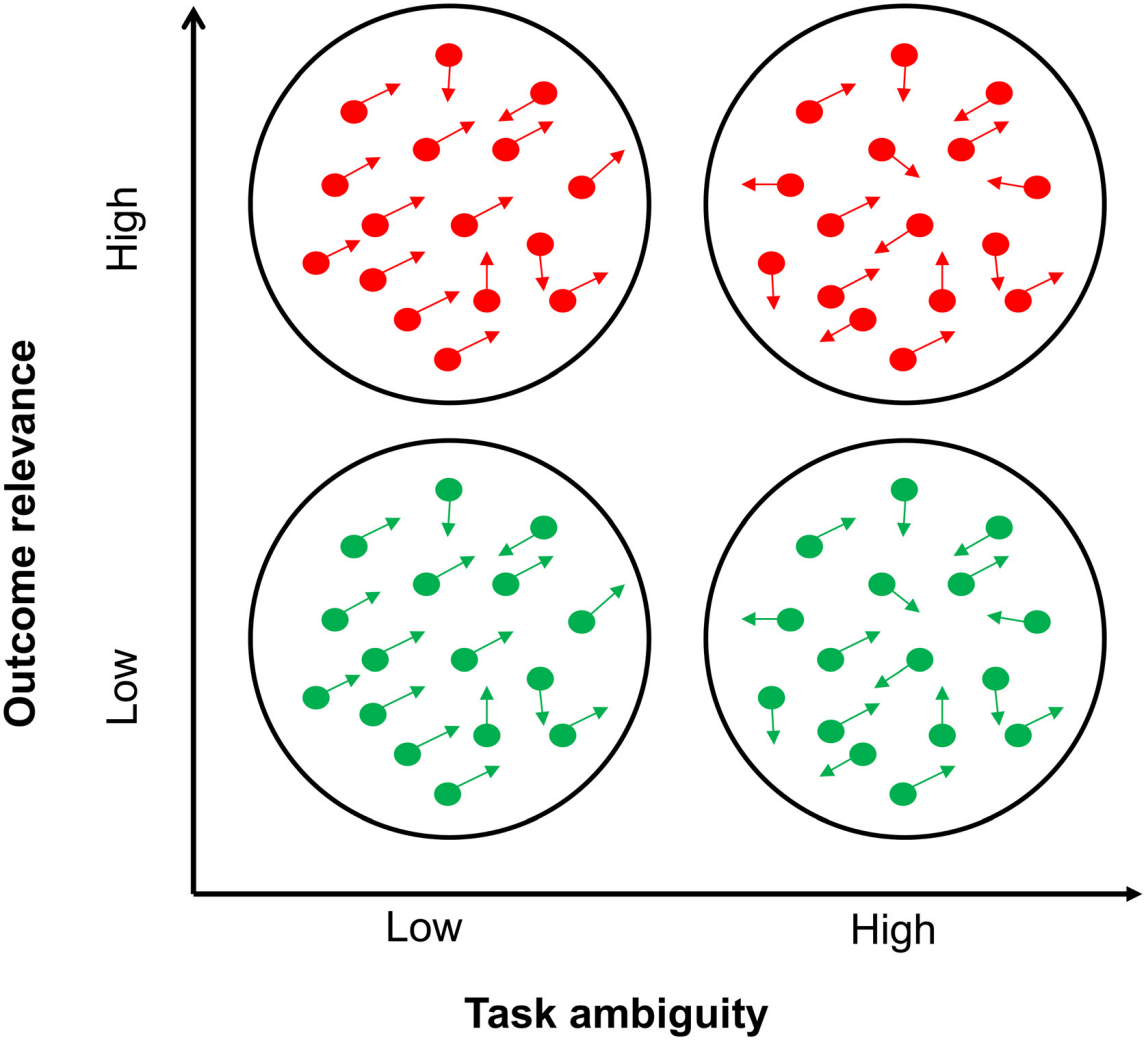
Task ambiguity and outcome relevance. Schematic representation of the two independent variables manipulated during the decision task: outcome relevance and task ambiguity. Low outcome relevance trials (no points at stake) were indicated by green dots; high outcome relevance trials (30 points at stake) were indicated by red dots.

In half the trials the dots in the RDM stimulus were depicted in green and in the other half they were depicted in red. Volunteers were told that the color indicated the reward available for correct performance. When the dot cloud was green there were no points at stake (low outcome relevance), whereas when the dot cloud was red they could win or lose 30 points depending on their performance (high outcome relevance). This resulted in a 2 by 2 factorial design, with task ambiguity and outcome relevance as the main within-subjects factors (Fig 2).

A 1000 ms presentation of a colored static cloud was used at the start of each trial to inform the subject of the outcome relevance of that trial. In the following 5000 ms the dots moved along the designated trajectory at the designated coherence level. During this phase subjects were required to indicate the overall direction of dots by pressing either the left or the right key.

Subjects were told that only the participant with the highest final score would have get a financial bonus of €10 but at the end of the experiment all participants were compensated with €10 and debriefed about the purpose of the experiment.

### 2.5. Statistical Analyses

Two one-way ANOVAs with NCC group (low; medium; high) as between-subjects factor and motion coherence level as a dependent variables were used to confirm that there were no group differences in motion coherence levels.

Next we assessed the linear relationship between accuracy and RT (i.e. the speed-accuracy trade-off effect [50]) by linear correlation analysis of mean accuracy and RT data (averaged across task ambiguity and outcome relevance factors) for the sample as a whole.

Finally we investigated the hypothesized moderation of the associations between cognitive effort and task ambiguity and outcome relevance by NCC, using a mixed ANOVA with NCC group (low; medium; high) as between-subjects factor and outcome relevance (low; high) and task ambiguity (low; high) as within-subjects factors and RT as the dependent variable.

## Results

### 3.1. Psychophysical Calibration

The two one-way ANOVAs on the selected motion coherence levels in the three NCC groups showed no significant group differences (50% level: *F*(1,52) = .352; *p* = .705; 25–75% levels: *F*(1,52) = .985; *p* = .382)

Analysis of the correlations between RT and accuracy indicated that there was no relationship between speed and accuracy, indicating that subjects’ RTs were a good index of the effort invested in the task and were unrelated to the accuracy of their performance (low outcome relevance + low task ambiguity: *r* = .027; *p* = .846; high outcome relevance + low task ambiguity: *r* = -.112; *p* = .420; low outcome relevance + high task ambiguity: *r* = .010; *p* = .940; high outcome relevance + high task ambiguity: *r* = .156; *p* = .259).

### 3.2. Main Experiment

A 3 (NCC: low; medium; high) x 2 (outcome relevance: low; high) x 2 (task ambiguity: low; high) ANOVA revealed main effects of NCC (*F*(1,52) = 4.360; *p* = .018), outcome relevance (*F*(1,52) = 7.682; *p* = .008; partial η^2^ = 1.141), and task ambiguity (*F*(1,52) = 31.412; *p* = .001; partial η^2^ = .376). Interestingly there were also interactions between NCC and outcome relevance (*F*(1,52) = 3.266; *p* = .046; partial η^2^ = .120) and between NCC and task ambiguity (*F*(1,52) = 3.671; *p* = .032; partial η^2^ = .116). The interaction between outcome relevance and task ambiguity was instead not significant (F(1,52) = 0.712; p = .495; partial η2 = .024) and there was also a lack of NCC x task ambiguity x outcome relevance interaction (F(1,52) = 0.712; p = .495; partial η2 = .026).

**Fig 3:**
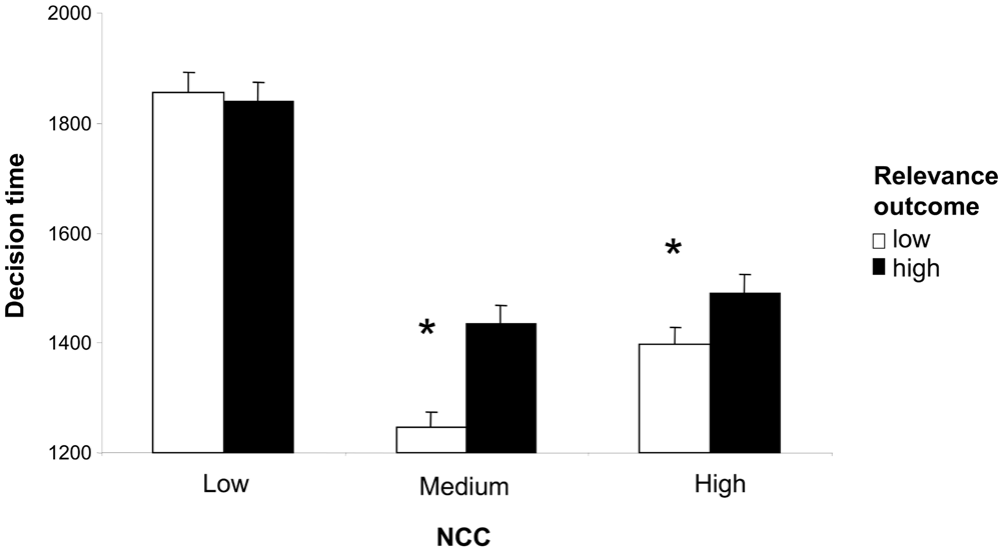
Interaction between NCC and outcome relevance. The graph shows mean RTs as a function of NCC and outcome relevance (low; high). The asterisks highlight the significant increases in cognitive effort (RTs) which occurred in trials with high outcome relevance in medium and high NCC subjects but not low NCC subjects.

**Fig 4:**
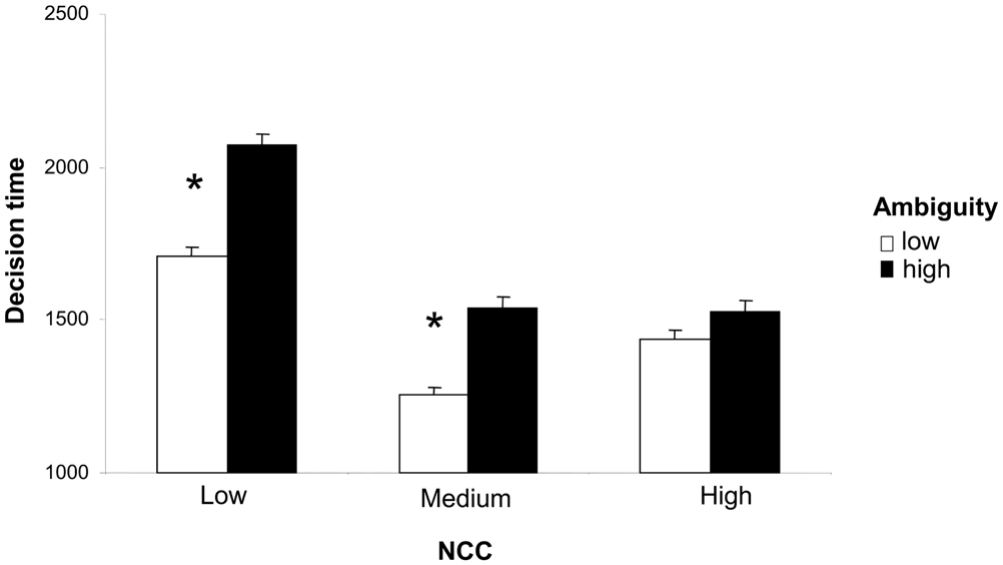
Interaction between NCC and task ambiguity. The graph shows mean RTs as a function of NCC and task ambiguity (low; high). The asterisks highlight the significant increase in cognitive effort (RTs) which occurred in high ambiguity trials in low and medium NCC subjects but not high NCC subjects.

The existence of a main effect of NCC confirmed the prediction that participants with low NCC would invest more cognitive effort in the perceptual task. Post-hoc comparisons of RTs in the three NCC groups indicated that mean RTs were higher in the low NCC group than the medium or high NCC groups (*p* < .05). The efficacy of the two experimental manipulations was confirmed by the effects of outcome relevance and task ambiguity on RTs. The three main effects were, however, qualified by two interactions, between NCC and outcome relevance (Fig 3) and between NCC and task ambiguity (Fig 4). Newmann-Keuls post-hoc analysis confirmed that high outcome relevance only increased RTs in medium and high NCC participants (low vs. high outcome relevance, medium NCC: *p* = .001; high NCC: p = 0.005; low NCC: *p* = .702). Task ambiguity showed the opposite pattern of association with post-hoc analyses confirming that high task ambiguity only increased RTs in low and medium NCC participants (low vs. high ambiguity, low NCC: *p* = .004; medium NCC: *p* = .001; high NCC: p = .400).

## Discussion

This study provides new insight into the factors which influence the level of cognitive effort invested in a task by demonstrating that their influence can be moderated by individual differences in NCC. In particular, we showed that the effect of changes in task difficulty varies according to dispositional NCC: low NCC individuals increased their cognitive effort when the discrimination was more difficult but high NCC individuals did not. Task ambiguity alone is not sufficient to prompt high NCC individuals to increase their cognitive effort.

A different pattern of results was seen when outcome relevance effects were analyzed. High NCC individuals invested more cognitive effort when correct performance resulted in monetary gain whereas outcome relevance did not affect the performance of low NCC individuals. This pattern of results is reminiscent of the distinction between intrinsic and extrinsic motivation (e.g. Self-Determination Theory, [25]) and suggests that low NCC subjects—assumed to be intrinsically motivated to determine the direction of the dot cloud—invested more cognitive effort to cope with the increase in task ambiguity whereas high NCC subjects—assumed to be extrinsically motivated—invested more cognitive effort only when correct performance was financially remunerative. This confirms and extends the Webster and Kruglanski [18]’s hypothesis of a negative association between need for cognition and need for closure as we found that only certain factors (in this experiment, availability of financial reward) were effective in increasing cognitive effort in individuals with high NCC. We conclude from these results that although people with high NCC generally exert less cognitive effort on a task, specific contexts lead them to increase their cognitive effort. The moderating effect of NCC on the relationships between cognitive effort and task ambiguity and outcome relevance which was observed in this study is consistent with previous research by Roets and colleagues [23] on the effects of situation-induced changes in NCC, and with complementary research by Westbrook and colleagues [2] on trait need for cognition [51], which has been shown to be moderately negatively associated with NCC [18].

In addition, the evidence that high NCC subjects invested more cognitive effort in conditions of high outcome relevance suggests that participants with high NCC levels are more motivated by the outcome. More specific investigation of cognitive effort using a design which manipulates exhaustion of cognitive resources in groups varying in dispositional NCC would be required to understand these relationships in detail.

A recent functional magnetic resonance imaging (fMRI) study [21] provided evidence on the neural correlates of NCC showing that high NCC was associated with reduced online adjustment in cognitive control, as indexed by adaptation to behavioral conflict. The same study showed that the difference in cognitive adaptation between high and low NCC participants was mediated by differences in the level of functional integration between two regions of the frontal cortex (the inferior frontal gyrus and dorsolateral prefrontal cortex) which are critically involved in top-down inhibitory control [52]. It is interesting to note that the same regions have also been associated with reduced self-control in the context of cognitive depletion [29].

Finally, it is worth noting that both manipulations affected the performance of the medium NCC group. We speculate that manipulations’ effects on medium NCC depend totally on the context, i.e. individuals with a medium NCC may invest more cognitive effort when confronted with highly ambiguous tasks and when task performance is associated with a monetary gain (outcome relevance).

These results have implications for theory development and for applications. Firstly they suggest that general rules describing how external, objective variables, such as stimulus ambiguity and outcome relevance, influence effort should be refined to take into account interactions with individual-level psychological factors such as NCC. Secondly, future research could explore whether NCC interacts with other variables that have been shown to affect the level of cognitive effort invested in a task, such as reward appraisal processes (see e.g. [53]). Unconscious reward cues increase cognitive effort without affecting the speed-accuracy trade-off or self-focus [54]. Thirdly, it would be interesting to assess inter-individual variability in NCC in children and to investigate whether NCC is involved in learning disabilities.

These result also have implications for work in contexts or on tasks that require intense or prolonged cognitive effort, for example they suggest that in these circumstances employers could maximize performance by selecting low NCC individuals to perform the tasks or by providing high NCC employees with an appropriate financial incentive.

Individual differences in motivation to invest cognitive effort should also be considered when making diagnostic assessments of neurocognitive abilities in order to avoid confusing low motivation with limited capability. In further investigations it would be interesting to use a combination of indices of cognitive effort (e.g. indices based on self-reported stress) or to administer this decision making task to a sample with a wider age range i.e. to evaluate potential age differences in response to manipulations of variables such as task ambiguity and outcome relevance.
